# Predictors for detecting chronic respiratory diseases in community surveys: A pilot cross-sectional survey in four South and South East Asian low- and middle-income countries

**DOI:** 10.7189/jogh.11.04065

**Published:** 2021-10-30

**Authors:** Dhiraj Agarwal, Nik Sherina Hanafi, Ee Ming Khoo, Richard A Parker, Deesha Ghorpade, Sundeep Salvi, Ahmad Ihsan Abu Bakar, Karuthan Chinna, Deepa Das, Monsur Habib, Norita Hussein, Rita Isaac, Mohammad Shahidul Islam, Mohsin Saeed Khan, Su May Liew, Yong Kek Pang, Biswajit Paul, Samir K Saha, Li Ping Wong, Osman M Yusuf, Shahida O Yusuf, Sanjay Juvekar, Hilary Pinnock, RESPIRE Collaboration

**Affiliations:** 1 Vadu Rural Health Program, KEM Hospital Research Centre (KEMHRC), Pune, India; 2 Faculty of Medicine, University of Malaya (UM), Kuala Lumpur, Malaysia; 3 Edinburgh Clinical Trials Unit, Usher Institute, The University of Edinburgh, Edinburgh, UK; 4 Pulmocare Research and Education (PURE) Foundation, Pune, India; 5 Hospital Pusrawi Sdn. Bhd., Kuala Lumpur, Malaysia; 6 School of Medicine, Taylor's University, Subang Jaya, Malaysia; 7 Christian Medical College (CMC), Vellore, India; 8 Bangladesh Primary Care Respiratory Society (BPCRS), Khulna, Bangladesh; 9 Child Health Research Foundation (CHRF), Dhaka, Bangladesh; 10 The Allergy & Asthma Institute (AAI), Islamabad, Pakistan; 11 NIHR Global Health Research Unit on Respiratory Health (RESPIRE), Usher Institute, The University of Edinburgh, Edinburgh, UK

## Abstract

**Background:**

Our previous scoping review revealed limitations and inconsistencies in population surveys of chronic respiratory disease. Informed by this review, we piloted a cross-sectional survey of adults in four South/South-East Asian low-and middle-income countries (LMICs) to assess survey feasibility and identify variables that predicted asthma or chronic obstructive pulmonary disease (COPD).

**Methods:**

We administered relevant translations of the BOLD-1 questionnaire with additional questions from ECRHS-II, performed spirometry and arranged specialist clinical review for a sub-group to confirm the diagnosis. Using random sampling, we piloted a community-based survey at five sites in four LMICs and noted any practical barriers to conducting the survey. Three clinicians independently used information from questionnaires, spirometry and specialist reviews, and reached consensus on a clinical diagnosis. We used lasso regression to identify variables that predicted the clinical diagnoses and attempted to develop an algorithm for detecting asthma and COPD.

**Results:**

Of 508 participants, 55.9% reported one or more chronic respiratory symptoms. The prevalence of asthma was 16.3%; COPD 4.5%; and ‘other chronic respiratory disease’ 3.0%. Based on consensus categorisation (n = 483 complete records), “Wheezing in last 12 months” and “Waking up with a feeling of tightness” were the strongest predictors for asthma. For COPD, age and spirometry results were the strongest predictors. Practical challenges included logistics (participant recruitment; researcher safety); misinterpretation of questions due to local dialects; and assuring quality spirometry in the field.

**Conclusion:**

Detecting asthma in population surveys relies on symptoms and history. In contrast, spirometry and age were the best predictors of COPD. Logistical, language and spirometry-related challenges need to be addressed.

Chronic Respiratory Diseases (CRD), most commonly asthma and chronic obstructive pulmonary disease (COPD), but also post-tuberculosis (TB) lung disease, bronchiectasis, interstitial lung disease and lung cancer, are common public health problems, with high prevalence and mortality rates globally, especially in low- and middle-income countries (LMICs) [1-4]. Symptoms such as cough, phlegm, shortness of breath, chest tightness and wheeze, are disabling features of CRD that contribute to poor health-related quality of life [5], impact on family, work and societal roles, as well as using health care resources.

Despite the high morbidity and mortality of CRD, awareness in LMICs is often low [6,7], with limited robust data on the true burden of disease in these countries [8]. Interpretations are fuelled by debates over spirometric thresholds and predictive value of (largely non-specific) respiratory symptoms [9]. Our previous scoping review of 281 CRD prevalence studies in LMICs identified several limitations that need to be addressed in future surveys [10]. These included:

1. Many surveys focused on detecting one condition (asthma or COPD); a few identified both, but hardly any mentioned other CRDs;
2. Algorithms for making a clinical diagnosis (as opposed to recording lung function) were often not well formulated, especially for asthma;
3. Restrictive lung conditions were rarely reported;
4. The impact of CRD on the quality of life of individuals, or their social and healthcare burden were rarely reported.

To understand how to resolve these evidence gaps, we conducted the Four-Country ChrOnic Respiratory Disease (4CCORD) pilot cross-sectional survey in five sites across four South/South-East Asian LMICs that were members of the NIHR Global Health Research Unit on Respiratory Health (RESPIRE) collaboration [11]. The aim was to: 1) use all available information (survey, spirometry and clinical reviews and expert opinion) to reach consensus on a clinical diagnosis (asthma, COPD or ‘other CRD’); 2) identify variables that predict the clinical diagnoses of asthma or COPD, and 3) describe practical barriers and solutions to undertaking the survey in South/South-East Asian LMIC contexts.

## METHODS

### Ethical approval

The cross-sectional survey was conducted in 2019 with local ethics approvals from respective Institute’s Ethics Committee or Review Board: Bangladesh Institute of Child Health Review Board BICH-ERC-02-06-2018 (Bangladesh); King Edward Hospital Research Center Ethics Committee: KEMHRC/RVM/EC/1191 (KEMHRC, India); Christian Medical Centre Institutional Review Board: IRB:11382 (OBSERVE) (CMC, Vellore, India) and Health Ministry’s Screening Committee, Government of India: 2018-0968; Medical Research & Ethics Committee: NMRR-18-2923-42961 (IIR) (Ministry of Health, Malaysia); Institutional Review Board, International Research Force: IRFIRB042019 (Islamabad, Pakistan)]. The study was sponsored by the University of Edinburgh (ACCORD: Reference number: AC18111). Written informed consent was provided by all study participants.

### Study sites and population

We recruited a random selection of 100 adults (18 years or over) at each of the five sites in four countries [Bangladesh (1); India (2); Malaysia (1); Pakistan (1)] for this cross-sectional survey. As this was a pilot study, we did not perform any formal sample size calculation. The characteristics of each site and the arrangements used to identify and recruit the participants are detailed in **Table 1**. In summary, all sites used computer-generated random numbers, but Bangladesh and the two sites in India sampled from their existing Health and Demographic Surveillance System (HDSS) databases, whilst Malaysia and Pakistan used a staged process of randomly selecting first the areas/quarters, then (in Malaysia) the households, followed by individuals.

**Table 1:** Characteristics of sites and arrangements for the survey

Country (site, locality)	Characteristics	Random sampling strategy	Language, translations of survey tools
**Bangladesh,** Child Health Research Foundation (CHRF), Mirzapur Upazila, n = 100	Rural sub-district located 65 km north of Dhaka	Computer generated random sample of adults residing in Mirzapur Upazila from Demographic Surveillance System (DSS) database	Although there had been a BOLD-1 site in Dhaka, the Bangladesh site was unable to obtain the Bengali version. The site used forward and backward translation process to translate the English questions to Bengali language.
**India,** Christian Medical College (CMC), Vellore, n = 101	Rural site located 25 km from Vellore town.	Computer generated random sample of adults residing in 18 rural Peripheral Service Units in the Rural Unit for Health and Social Affairs (RUHSA) population database.	The BOLD questionnaire is available in Tamil language. However, the local dialect used in the rural site is significantly different from standard Tamil, so the researchers had to explain/adapt specific words to ensure the questionnaire was understood by local communities
**India,** KEM Hospital Research Centre, Pune, n = 106	Rural site located at Manchar 70 km from Pune city.	Computer generated random sample of adults residing in the Junnar block in the Health and Demographic Surveillance System (HDSS) database	The BOLD questionis available in Marathi language, and had been translated locally and used previously when the site contributed to the BOLD study.
**Malaysia,** University of Malaya (UM), Kuala Lumpur, n = 101	Urban; Klang District	The Department of Statistics Malaysia randomly selected 200 Living Quarters in the Klang District, randomly sampled one household within each quarter, and then randomly selected one member of the household to be surveyed to a total of 101 participants	The site used the English and Malay versions, as preferred by the participant. The BOLD questionnaire was available in Malay language, but there were concerns about local appropriateness of the translation. The site used forward and backward translation process to translate the English questions to the Malay language.
**Pakistan,** Allergy & Asthma Institute (AAAI), Islamabad, n = 100	A mix of urban Islamabad and surrounding rural areas	Randomly selected areas within Islamabad and Rawalpindi and then randomly selected adults from the population census lists of those areas	The BOLD questions are available in Urdu language. However, the local dialect used is significantly different from standard Urdu, so the researchers had to explain/adapt specific words to ensure the questionnaire was understood by local communities.

*The RESPIRE study questionnaire was adapted with permission from BOLD-1 [12] with the addition of eight asthma-related questions from ECRHS-II [13].

#### Study questionnaire and clinical algorithm development

In February 2019, the investigators from all five study sites and the University of Edinburgh conducted a workshop to determine the survey procedures. Informed by the preliminary findings of the scoping review, a range of validated questionnaires that had previously been used in LMICs to detect CRD were considered from the perspective of the conditions that we wished to identify and availability of local language versions. We decided to use the widely used BOLD-1 questionnaire (v3.1) [12], which detected COPD and other respiratory symptoms and asked about TB and co-morbidities. The BOLD questionnaire (v3.1) was available in all languages used in these sites though problems obtaining/using the existing version meant that two sites (Bangladesh and Malaysia) undertook a new forward/backward translation (see **Table 1**). We added eight questions from the ECRHS-II questionnaire to detect asthma [13], and some sites had to translate these questions to their local language. For clarity, we refer to the final survey tool as the ‘RESPIRE study questionnaire’.

### Spirometry

We performed spirometry using calibrated EasyOne Air spirometers (NDD Medical Technologies Inc, Andover MA, US). Reversibility was tested 15 minutes after administration of salbutamol 400μg (via metered dose inhaler and spacer). Study staff from each site were trained by the team from the Pulmocare Research and Education (PURE) Foundation, Pune, India, to conduct spirometry according to the American Thoracic Society (ATS) and European Respiratory Society (ERS) guidelines [14]. Quality checks of the spirometry data generated were performed by SS and DG (Senior respiratory physician and Spirometry Trainer at the PURE Foundation, Pune, India). Height and weight of the study participants were assessed by trained field workers using calibrated stadiometers and adult weighing scales to a precision of 0.1 cm or 0.1 kg, respectively. Age and ethnicity were self-reported by the participants. Age was recorded in completed years as the exact birth dates for some older participants were not available. We used Global Lung Function Initiative (GLI) 2012 reference values to calculate predicted values and lower limit of normal FEV_1_/FVC to define obstruction [15]. We used ethnicity as “Southeast Asian” for the Malaysian data set and “Other or Mixed” for the Bangladesh, Indian and Pakistan sites from the options in the online GLI calculator (http://gli-calculator.ersnet.org/).

### Data collection and entry

Data were collected at each site using interviewer-administered hard copies of the RESPIRE study questionnaire (resource limitations precluded development of bespoke data entry software in this pilot study). All data were entered into Microsoft Excel by the data entry operators. Spirometry data printouts with graphs were available for quality check and clinical assessment.

### Clinical assessment

Anonymised data were shared with SS or DG who assessed the spirometry for quality and provided a provisional diagnosis based on spirometry and RESPIRE study questionnaire responses. SS/DG identified a list of participants in whom further information would be useful to clarify the diagnosis (specifically those with borderline or unclear spirometry, or where spirometry and symptoms were mismatched) and these participants were invited to a clinical examination with a local pulmonologist (or in Malaysia a family medicine specialist supported by a pulmonologist). The purpose of the clinical review was explained to the clinicians who were instructed to complete a clinical record sheet to document medical history, examine the participant and, at their discretion, repeat the spirometry or arrange any additional tests (See Appendix S1 in the **Online Supplementary Document**). All clinical findings were documented and the likely diagnosis recorded on a paper record sheet that was scanned and added to the study documentation.

### Consensus diagnosis categorisation

Participants were categorised into one of ten diagnostic categories (defined in **Table 2**) determined by consensus of three primary care physicians with expertise in respiratory disease (NSH, EMK, HP). Each physician independently determined the diagnostic category based on the RESPIRE study questionnaire responses, spirometry print-outs, spirometry quality reports (from SS/DG) and the examining physician’s report (for selected participants). Disagreements were discussed, discrepancies resolved, and agreement reached between all three physicians. The consensus decision was then considered as the ‘gold-standard’ diagnosis.

**Table 2:** Diagnostic categories

Diagnostic category	Description	Gold-standard diagnosis	N = 508
COPD	COPD based on obstructive spirometry (CRD symptoms and FEV_1_/FVC<LLN). Clinical discretion was allowed if FEV_1_/FVC fell between LLN and fixed ratio of 70% according to symptoms/risk factors.	COPD	23
Asthma (spirometry)	Asthma based on spirometry: obstructive spirometry with substantial BD reversibility (increase in FEV_1_ of >15% and >400 mls) (19)	Asthma	8
Asthma (symptoms)	Asthma based on a number of symptoms, self-reported physician diagnosis, atopic co-morbidities, and family history: spirometry normal		75
Other CRD	Other Chronic Respiratory Disease (post-TB, bronchiectasis/chronic bronchitis with normal spirometry)	Other CRD	15
RLD	Restrictive Lung Disease: restrictive spirometry (FVC<80% and FEV_1_/FVC>LLN) with one or more CRD symptom	RLD	65
No CRD	Asymptomatic and normal spirometry	No CRD	192
Isolated symptom (CRD unlikely)	Isolated symptom that could be due to CRD (usually asthma) but no other evidence of CRD and normal spirometry.	CRD unlikely	42
Restrictive (asymptomatic)	Restrictive spirometry (FVC<80% and FEV_1_/FVC>LLN) but asymptomatic	Asymptomatic restrictive	45
Non-respiratory	Symptoms likely to be due to a non-respiratory cause (eg, heart disease; anaemia)	Non-respiratory	28
Unclear	Unclear symptoms; uninterpretable spirometry	Unclear	12

COPD – chronic obstructive pulmonary disease, CRD – chronic respiratory disease, FEV_1_ – forced expiratory volume in 1 s, FVC – forced vital capacity, LLN – lower limit of normal, BD – bronchodilator, RLD – restrictive lung disease

### Data/Statistical analysis

We performed the analysis using the Stata v15 software,R software version 3.5.3[16], and SAS software version 9.4 (SAS Institute Inc., Cary, NC, USA). Descriptive statistics were performed for all socio-demographic and ‘gold standard’ diagnosis categories.

Logistic regression with Lasso model selection (based on the Schwarz Bayesian Criterion) was conducted using the HPGENSELECT procedure within SAS software version 9.4 (SAS Institute Inc., Cary, NC, USA) and was used to identify predictors of the “gold-standard” diagnoses of (i) Asthma (symptoms/spirometry), and (ii) COPD. This method was used rather than standard modelling methods to minimise the risk of overfitting and increased the predictive ability of our models [17]. The variables shown in Table S1 of the **Online Supplementary Document** from the RESPIRE study questionnaire and spirometry reports were all included as explanatory variables in the Lasso regression models. We then used an internal validation method (bootstrapping) to validate the fitted regression models in R software version 4.0.4 [16,17]. This involved bootstrapping the participant rows of the data, and then calculating the predicted values for each bootstrap data set using the original fitted models. A calibration slope was then calculated for each bootstrap data set via logistic regression of the outcome in the bootstrap data set with the predicted values as the single explanatory variable [17]. A mean calibration slope was then calculated for each fitted model, with a value close to one indicating a model with good calibration [17].

The parameter estimates from the lasso regression model for asthma were used to calculate a risk score for diagnosing asthma. This involved calculating the sum of all relevant coefficients (except the intercept term) to calculate a diagnostic score R. For converting the risk score R to calculate a probability of asthma diagnosis, the probability of asthma P is *P* = *exp* (R + I) / [1 + *exp* (R + I)], where R is the risk score, I is the intercept term, and *exp* is the exponential function.

A Receiver Operator Characteristic (ROC) curve was constructed based on the model predicted values (risk score) derived from the lasso regression using the ROCit package [18] in R software. We also calculated the area-under-the-curve (AUC) of the ROC curve with 95% confidence intervals.

### Experiences and challenges of conducting the survey

To assess feasibility, each recruiting site documented their experiences and challenges during the preparatory phase, survey administration and conduct of the study. Major concerns or hurdles were collated for reporting, and any remedial action described.

### Patient and public involvement

Community Engagement and Involvement has been a core activity in all centres of the RESPIRE collaboration. Participants and other stakeholders have been actively engaged in developing the RESPIRE research agenda, reviewing proposals, and are now involved in disseminating the findings of projects.

## RESULTS

The socio-demographic characteristics of the 508 participants surveyed from the five sites are given in **Table 3**. Note that differences in characteristics between the sites (eg, younger participants in Pakistan) may be explained by the small sample sizes in this feasibility study. 177 (34.8%) had a clinical assessment. Overall, 283/508 (55.7%) reported one or more chronic respiratory symptoms, most commonly breathlessness on walking uphill (25%) or wheezing (21%). 33 (6.5%) reported breathing problems that interfered with daily activities.

**Table 3:** Characteristics of the study population*

Site: country (institute)	Sex: male n (%)	Age: mean years (SD)	BMI: mean (SD)	Ever smoked, n (%)†	Dusty job, n (%)‡	Biomass cooking, n (%)§	≥1 CRD symptom, n (%)
Bangladesh (CHRF) N = 101	43 (43)	44.7 (14.6)	24.6 (4.5)	28 (28)	71 (70)	90 (89)	51 (51)
India (CMC) N = 100	42 (42)	43.6 (10.8)	25.4 (4.9)	9 (9)	77 (77)	95 (95)	36 (36)
India (KEMHRC) N = 106	52 (49)	41.8 (16.3)	22.3 (4.0)	5 (5)	14 (13)	75 (71)	50 (47)
Malaysia (UM) N = 101	51 (50)	44.0 (14.5)	25.7 (5.2)	30 (30)	33 (33)	0 (0)	65 (64)
Pakistan (AAI) N = 100	57 (57)	36.3 (13.4)	25.9 (6.3)	28 (28)	19 (19)	16 (16)	82 (82)

BMI – body mass index, SD – standard deviation, CRD – chronic respiratory disease, CHRF – Child Health Research Foundation, CMC – Christian Medical College, KEMHRC – King Edward Memorial Hospital Research Centre, UM – University Of Malaya, AAI – The Allergy & Asthma Institute

*The differences in characteristics between the sites are likely to be due the small sample sizes in this feasibility study rather than real differences in populations that would have been captured in a fully powered survey with approximately 1000 participants/site.

†‘Ever smoked’ is a positive response to Q1028: Have you ever smoked cigarettes?

‡‘Dusty job’ is a positive response ‘to Q1034: Have you ever worked for a year or more in a dusty job?

§^‘^Biomass cooking’ is a positive response to Q1057: Has an indoor open fire with wood, crop residues or dung been used as a primary means of cooking in your home for more than 6 mo in your life?

### ‘Gold-standard’ diagnostic categorisation

**Figure 1:**
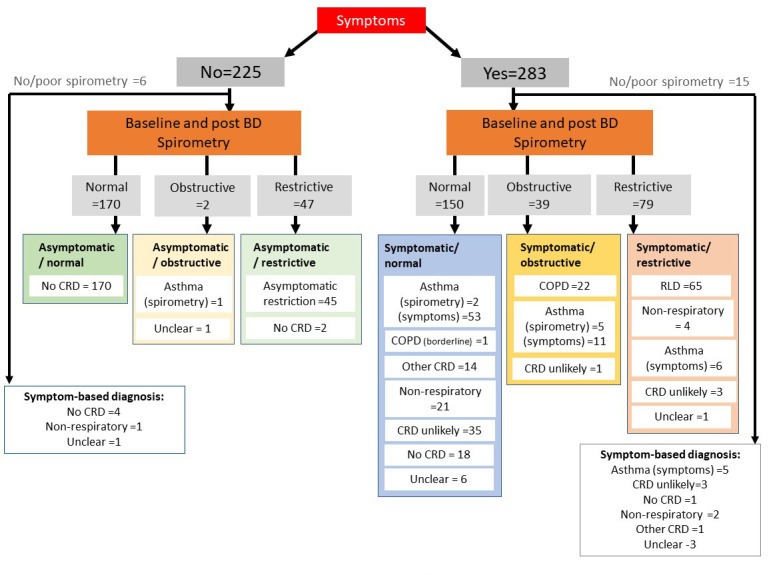
Clinical diagnostic algorhitm.

These are illustrated in the clinical diagnostic algorithm (**Figure 1**), and listed in **Table 2**.

In 12 of the 508 cases, no diagnostic category could be determined (mostly due to uninterpretable spirometry and/or inconsistent symptomatology) leaving 496 for analysis. Based on consensus categorisation, the prevalence of asthma was 16.3% (83/508), COPD was 4.5% (23/508); and ‘other CRD’ 3.0% (15/508). Non-respiratory causes (eg, heart disease/anaemia/obesity) were considered to be the cause of symptoms in 5.5% (28/508). Proportions varied by site, but as a pilot survey, we were underpowered to compare results by site, so these results are only provided in Table S2 of the **Online Supplementary Document**.

Of the 83 participants diagnosed with asthma, only eight had obstructive spirometry with substantial reversibility (>400mls/15% specified by GINA as ‘confidently’ confirming an asthma diagnosis); 75 had a combination of symptoms, atopic co-morbidities and/or were diagnosed/treated for asthma [19]. 42 had an isolated symptom that could have been due to asthma, but could have had other explanations (eg, a single episode of a cough disturbing sleep could be due to a viral respiratory infection). In the absence of other CRD symptoms or abnormal spirometry, these were described as ‘isolated symptom (CRD unlikely)’ and were not included in the asthma category for further analysis.

Based on GLI predictive values, 21.6% (110/508) had restrictive spirometry as defined by a post-bronchodilator FVC<80% and normal FEV_1_/FVC ratio. Of these, 45 were asymptomatic. At the KEMHRC site, 27/106 (25.0%) participants had restrictive spirometry based on GLI 2012 predictive values. This was reduced to 13 (12.2%) when locally calculated predictive values were used [20].

### Predictors for asthma and COPD

Lasso regression showed different variables were predictive of asthma and COPD (ten for asthma and three for COPD). The final models (linear predictors) for asthma and COPD are shown in **Table 4**. The models were based on a sample size of N = 483 (out of the total 496 cases analysed, 13 were dropped due to missing data for the variables).

**Table 4:** Parameter estimates for a predictive models for asthma and COPD*

Asthma	Lasso predictive model estimate
Intercept	-2.6215
Age	-0.0090
Did not have wheezing in the last 12 months	-0.4217
Had wheezing in the last 12 months	0.3741
Never had trouble with breathing	-0.1881
Had trouble with breathing	0.1608
Not woken up with a feeling of tightness [ECRHS II]	-0.5401
Woken up with a feeling of tightness [ECRHS II]	0.4829
Not had an attack of SoB that came on following strenuous activity at any time in the last 12 months [ECRHS II	-0.3810
Had an attack of SoB that came on following strenuous activity at any time in the last 12 months [ECRHS II]	0.3354
Have not been woken by an attack of coughing at any time in the last 12 months [ECRHS II]	-0.2323
Have been woken by an attack of coughing at any time in the last 12 months [ECRHS II]	0.2006
No nasal allergy	-0.0085
Nasal allergy	0.0069
Pre-FEV_1_%pred	-0.0812
Post-FEV_1_%pred	0.0924
Post-FVC%pred	0.0125
**COPD**	**Lasso predictive model estimate**
Intercept	-0.5325
Age	0.0436
Post-FEV1%pred	-0.2313
Post-FVC%pred	0.1532

ECRHS – European Community Respiratory Health Survey, SoB – short of breath, COPD – chronic obstructive pulmonary disease, FEV_1_ – forced expiratory volume in 1 s; FVC – forced vital capacity

*Confidence intervals around the parameter estimates are not automatically generated in lasso regression; our focus was mainly on developing a reliable predictive model of asthma and COPD that could be used in future surveys.

### Predictors for asthma

Within this sample, 76 participants had asthma (ie, ‘asthma spirometry’ and ‘asthma symptoms’ according to gold-standard diagnosis) and 407 without asthma. The final logistic regression model of the gold-standard asthma diagnosis included ten distinct variables: “age”, “Did you have wheezing in the last 12 months?”, “Have you ever had trouble with breathing?”, “Woken up with a feeling of tightness” [ECRHS II], “Had an attack of shortness of breath (SoB) that came on following strenuous activity at any time in the last 12 months”, “Woken by an attack of coughing at any time in the last 12 months [ECRHS II]}”, nasal allergy, “pre-FEV_1_%pred”, “post-FEV1%pred””, and “post-FVC%pred”. Of these “Did you have wheezing in the last 12 months?”[BOLD I], “attack of shortness of breath following stenuous activity” [ECRHS II] and “Woken up with a feeling of tightness” [ECRHS II], were the strongest predictors of a gold-standard asthma diagnosis.

Our internal (bootstrap) validation procedure for the asthma model (see **Table 4**) generated an average calibration slope of 1.17, which was close to 1. This indicates that the model has good calibration and should perform well when fitted to a new set of participants in future large-scale surveys and generate accurate asthma diagnostic risk scores.

The parameter estimates from the lasso regression model for asthma (**Table 4**) were then used to calculate a risk score for diagnosing asthma. After omitting the intercept term, the diagnostic score R ranged from -0.56 to 3.48, with higher values indicating a greater probability of a gold-standard asthma diagnosis.

**Figure 2:**
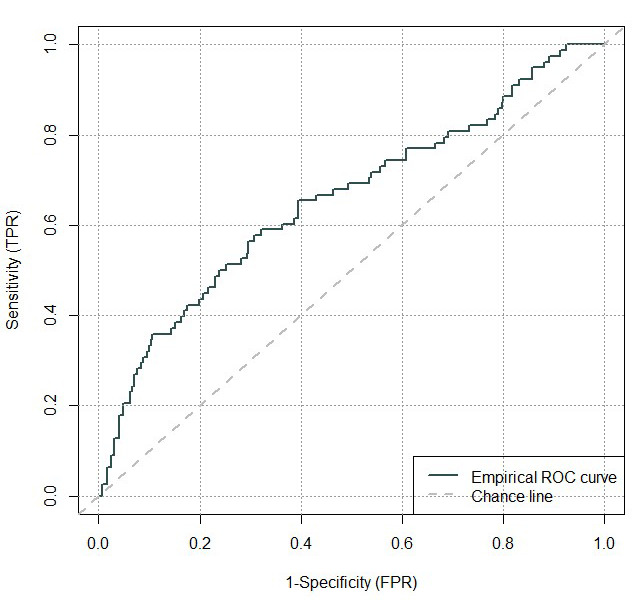
ROC curve showing sensitivity against specificity for model predicted values compared with the gold-standard asthma diagnosis.

A ROC curve was constructed for the diagnostic score R based on the parameters in **Table 4** (see **Figure 2**). The area-under-the-ROC curve was low 0.66 (95% confidence interval (CI) = 0.59 to 0.73), and there was no clear optimum cut-off point identified from the ROC curve. However, separate cut-off points can be found which ensure the sensitivity and specificity are above 90% and allow us to identify true asthma cases and true non-cases. Values of R above 2.33 were associated with a specificity of over 90%, allowing us to “rule in” an asthma diagnosis for all participants with R>2.33 in a future survey. Similarly, participants with R<1.35 are very unlikely to have asthma since the test sensitivity was over 90% for cut-off values below 1.35. In total, these two groups comprise approximately 31% of the overall study sample. However, there still remains 69% of participants with R scores in the range of 1.35 and 2.33 which it would have been difficult to classify, if only this diagnostic algorithm had been used.

### Predictors for COPD

Regarding COPD, 22 participants had a gold-standard COPD diagnosis and 461 did not. For COPD (n = 483), three distinct variables were included in the model: “age”, “post-FEV1%pred”, and “post-FVC%pred”. “Post-FEV_1_/FVC ratio” is moderately correlated with ‘age’ (correlation coefficient -0.43) and, after adjusting for the three included variables, was not significant and was therefore not included in the final model.

For the COPD model, the calibration slope was only 0.53. In a lasso regression model of COPD, including demographic and symptom questions and no spirometry (n = 496), only age appeared in the final model (parameter estimate 0.066).

### Distinguishing asthma from COPD

Fitting the same model to those with a diagnosis of asthma and COPD patients/participants only (n = 98), the same predictors appeared in the final model as in **Table 4**, with the addition of pre-FVC%pred (see Table S3 in the **Online Supplementary Document**). Age and spirometry are useful not only to distinguish between participants with COPD and those without, but also to distinguish between those with COPD and those with an asthma diagnosis.

### Feasibility and learnings from conducting the survey

**Table 5** lists the barriers and challenges noted by the five sites. One particular challenge faced was the difficulties with the existing versions of the study questions which the researchers reported having to explain in the local dialect to some participants.

**Table 5:** Barriers and challenges to conducting the survey

	Barriers and challenges as described by researchers
**Questionnaire development**	Some validated translations were unclear, and questions used expressions that needed translating into the local dialect or concepts that needed to be explained by researchers. Specifc examples include, the Tamil word for a ‘cold’ (viral upper respiratory infection) was corrected from ‘thadiman’ (which translates as ‘thickness’) to ‘jalathoṣam’. The word ‘Vaithiyar’ (used for unqualified ‘doctors’) was changed to ‘Maruthuvar’. In some dialects of Indian vernacular language there is no specific term for asthma, and in Malay the local term 'lelah' denotes both asthma and COPD.
**Maintaining quality of spirometry**	There were few existing trained spirometry technicians, so sites needed to train research assistants to conduct spirometry. Additional training was needed to maintain quality especially regarding importance of performing an inspiratory loop.
	Turnover of research assistants necessitated repeated training
**Barriers to data collection**	Variable working hours of potential participants on weekdays meant that surveys needed to be conducted in evenings/weekends.
	Data collection coincided with Ramadan making it difficult to recruit in Muslim communities
	Language barriers when communicating with participants of different ethnicity (eg, in Malaysia which has three ethnic groups and languages) during recruitment may have led to participants’ refusal.
	Cultural norms (eg, the need to refer to the head of family for a decision to participate)
	Reluctance of participants to attend the clinic review
**Enumerator safety**	Safety was a concern in some areas with need for research assistants to work in groups
	Societal fear of crime, dogs, harassment
	Insurance for the research team was costly

COPD – chronic obstructive pulmonary disease

## DISCUSSION

Over half the participants in our survey reported at least one chronic respiratory symptom. Based on a consensus categorisation, we estimated the prevalence of asthma as 16.3%; COPD 4.5%; restrictive spirometry (based on GLI predictive values) 21.6%; and ‘other CRD’ 3.0%. 5.5% were considered to have non-respiratory causes for their symptoms. Of the 83 participants with asthma, only eight could be confirmed confidently with spirometry.

### Predicting an asthma diagnosis

The fitted model for diagnosing asthma showed good calibration and performed well in the internal validation, so could be used to give an estimated probability of asthma in full-scale surveys. Unfortunately however, the low area-under-the-ROC curve meant that we could not identify a single cut-off point that would enable classification of all participants into binary categories of ‘asthma’ or ‘not asthma’. However, the prediction model could determine sub-populations that are either highly likely to have asthma or highly unlikely to have an asthma diagnosis, though this only classified about a third of the cases we detected by clinical consensus.

### Predicting a COPD diagnosis

In contrast, COPD diagnosis appeared to be mainly dependent on spirometry. Questions based on respiratory symptoms did not appear to be helpful, only age was included in the final model with “post-FEV_1_%pred”, and “post-FVC%pred”. The (clinically surprising) omission of “post-FEV_1_/FVC ratio” is because the ratio is correlated with age. However, the models cannot be relied upon for predictive purposes as they showed poor calibration and exhibited substantial overfitting, even after using lasso regression. We suspect this was because of the low event rate for COPD in our study. The variables we identified as potential predictors of COPD need confirmation in future studies.

### Strengths and limitations

This was a pilot cross-sectional survey, and the small numbers at each site mean that we were not powered for detecting prevalence in individual countries, though our data may be a guide for sample size calculations in future full-scale surveys. Notably, there were some differences in baseline characteristcs between sites that were likely to be due the small sample sizes in this feasibility study (100 participants/site) rather than real differences in populations that would have been captured in a fully powered survey with approximately 1000 participants/site.

The ‘gold-standard’ categorisation was achieved with as much rigour as possible (three physicians independently making a diagnosis based on a range of available raw data (survey responses, spirometry with graphs, and a clinical history/examination/tests from a clinician in 177 cases). Despite this, there may be some misdiagnoses, especially in categorising isolated symptoms when, despite consensus discussion, we could neither confirm nor conclusively rule out asthma (or other respiratory causes) in 45 participants.

We limited our survey to adults, so we cannot comment on the challenge of detecting chronic respiratory disease in children.

We intended to use existing translations of the widely used questions, so our findings aligned with major global surveys, though researchers reported difficulties with the ‘official’ or previously used translations which needed to be explained (or in two sites translated) into local dialects (see **Table 5** for examples).

Finally, the models developed have not been externally validated in other populations.

### Interpretation and implications for future surveys

Despite a sample size commensurate with a pilot survey, our finding that half our participants reported at least one chronic respiratory symptom, and a quarter were categorised as having a CRD suggests a significant burden of disease in the populations surveyed. These data were collected before the pandemic so were not affected by the rise in respiratory symptoms due to COVID-19. Other factors may be somatisation in contexts where mental health problems are stigmatised [21,22], and misunderstanding about translated words used to describe respiratory symptoms, diseases and health.

Most published surveys focus on one disease [10]. For example, in a recent systematic review, the global prevalence of COPD in adults 30-79 years was calculated as 7.7% (95% CI =  5.7-10.1) using the LLN-COPD definition (10.2% using FEV_1_/FVC<70%) [Adeloye D: personal communication]. Our estimate of 4.7% (using LLN) is lower, in part because our wide recruitment strategy included adults between 18 and 30 (unlikely to have significant COPD by virtue of age). In addition, this systematic review, in common with many surveys [10], equated obstructive spirometry with a diagnosis of COPD. In line with GOLD 2020 guidelines, we took a clinical perspective and only categorised a participant as having COPD if they reported at least one relevant symptom (shortness of breath, cough, phlegm) [23]. This will have overcome the ‘uneasy assumption’ in using FEV_1_/FVC<LLN, that COPD prevalence cannot be lower than 5% [24], as asymptomatic individuals with FEV_1_/FVC<LLN would not be categorised as COPD (as illustrated in **Figure 1**, in which an asymptomatic participant with obstructive spirometry was characterised as ‘unclear’). The limitation of not including symptom status is recognised, and standard questionnaires, eg,, BOLD (v3.1) [12] include questions on shortness of breath, persistent cough and interference with activities which could be incorporated in definitions. However, these symptom questions were not included in our final model after lasso model selection, and their application would need to be established in a larger sample. Cough and phlegm (along with age and peak flow) are included in the COLA ‘low cost screening tool’ derived and validated in Uganda [25].

Detecting asthma in a survey is even more problematic. Our systematic review highlighted that the definitions of asthma used in surveys were usually based on symptoms or patient/participant-reported diagnosis and medication usage [10]. The Global Burden of Disease define asthma as ‘a doctor’s diagnosis and wheezing in the past year’ [24] and reported a global prevalence of 3.6% (3.2% to 4.0%). Had we used ‘patient/participant-reported doctor-diagnosed’, we would have identified a comparable prevalence of 21/508 (4.1%). Spirometry was unhelpful, with only 8/83 (9.6%) demonstrating an increase in FEV_1_ post-bronchodilation of >400mls (defined by GINA as enabling a ‘confident’ diagnosis) [19]. Even using the lower threshold of >12% and 200mls (which is compatible with COPD [23]) only 18/83 (21.6%) of the participants we categorised as asthma had a spirometry confirmed diagnosis. Our ‘gold-standard’ clinical diagnosis thus relied on symptoms and resulted in a prevalence of 16.3%, quadruple the GBD estimate. This reflects the recognised clinical difficulty of making a robust diagnosis of asthma in the absence of definitive tests, exacerbated in the context of a population survey because of the limited information available from a single assessment made when the participant may (or may not) be symptomatic.

Other causes of CRD are rarely reported in surveys [23]. A recent systematic review reported interstitial lung disease, pulmonary sarcoidosis and pneumoconiosis (but not bronchiectasis, post-TB lung disease or cystic fibrosis) as having a very low prevalence of 0.07% [our unpublished results ]. Our clinical ‘gold-standard’ identified 3.0% as having symptoms probably due to CRD that was not asthma or COPD. Whilst further investigations would be required to establish the precise diagnoses, it is likely that these conditions are commonly overlooked in epidemiological studies and public health planning.

A fifth (21.6%) of the spirometry was classified as ‘restrictive’ with FVC<80% and FEV1/FVC>LLN when using GLI 2012 normal values; 41% of whom were asymptomatic. This is likely to be an artefact of using incorrect normal values. Reclassifying the spirometry from one of the sites with normal values for the Western Indian population [20], reduced the proportion of restrictive spirometry from 33.0% to 12.2%. In countries where normal values are not well defined, surveys should recruit enough participants to enable calculation of normal values from asymptomatic individuals. Evolving understanding of this non-specific restrictive spirometry – recently designated as ‘preserved ratio-impaired spirometry’ (PRISm) suggests that about a quarter will progress over time to diagnosed respiratory disease [26]. Risk factors for PRISm applicable to our population include post-TB and biomass fuel exposure. A prevalence of 12.2% is comparable to that described in other studies [26,27].

We faced a number of practical challenges, including a concern that the available translations of the questionnaires needed to be explained in the local dialect. Quality control is important in training for performing spirometry, with on-going oversight to maintain standards [28]. Future studies should take into account the timing of data collection and adapt to the local context, such as not recruiting during Ramadan or on weekdays, when recruitment was difficult. Engaging communities and village leaders would help to facilitate recruitment process by advising on timing, and improving access.

## CONCLUSION

Our consensus-derived gold-standard diagnosis enabled us to determine predictors of asthma, COPD, restrictive lung conditions, PRISm and ‘other CRD’ from the data collected in a questionnaire survey and spirometry at five sites in South/South East Asia. Detecting asthma in population surveys relies on symptoms and history and our findings may be used to derive predictive values for use in large-scale surveys. In contrast, spirometry is the basis for detecting COPD, possibly supported by participant demographic information. However, ensuring adequate sample size to determine local normal spirometry values is important. Despite the challenges of conducting surveys on CRDs in LMICs, accurate and reliable data on prevalence and impact on individuals, their families and communities are needed to inform health care policy on prioritising care and targeting risk factors such as smoking and poor air quality to reduce avoidable morbidity and mortality.

## Additional material


Online Supplementary Document
