# Supplementary material for: Predictors for detecting chronic respiratory diseases in community surveys: A pilot cross-sectional survey in four South and South East Asian low- and middle-income countries
Source: J Glob Health. 2021 Oct 30;11:04065. doi: 10.7189/jogh.11.04065 (PMC8561335; doi:10.7189/jogh.11.04065)
Supplement: Online Supplementary Document [file jogh-11-04065-s001.pdf]

**Appendix S1:** Instructions for clinicians undertaking clinical reviews

Participants had completed a screening questionnaire and undertaken spirometry. Dr Sundeep Salvi used the data that he has received from the survey + spirometry and categorized patients on this basis. He identified a list of participants in whom further information would be useful to clarify the diagnosis (specifically those with borderline or unclear spirometry, or where spirometry and symptoms were mismatched). He would send the list of participants needing clinical assessment to the Centres.

**The task in the clinical assessment is to reach a clinical decision about the diagnosis.**
You will have the results of the questionnaire survey and the spirometry results. What we need you to do is to use your clinical expertise to decide on clinical grounds what the diagnosis is – or if the patient is normal.

- Take a clinical history; examine the patient; if appropriate arrange further investigations (e.g. repeat spirometry to look for variability; ECG if you suspect a cardiac cause or if the pulse is irregular, CXR if the patient has haemoptysis, FBC if they may be anaemic). The decision to do additional tests is clinical – just as you would in a normal clinical consultation

**Please keep very clear notes.** Please complete the data collection form which has headings for these key components of a clinical assessment:

- History: What history did you take? What did the participant tell you about their symptoms, past history, family history? Any drug treatment?
- Examination: What examination did you do and what did you find? Specifically note findings of respiratory signs/chest examination, but also examination of other systems.
- Tests (if any): What tests did you do – and what was the result? Were there any investigations that might have helped but which were not available in your setting?
- Conclusion (your clinical opinion). What was your conclusion and why? If possible, indicate the cause of chronic respiratory symptoms, but it is understood that sometimes the diagnosis may not be clear – or the participant may have more than one problem
- Add any comments or feedback about the process/assessment

**In summary, what we need from the clinical assessment is your clinical expertise and your feedback on the process.**
It is important to remember that this is a feasibility study; your feedback will help us develop algorithms for the large scale 4CCORD survey. We will compare your expert opinion to the conclusion of the original survey/spirometry and assess the sensitivity/specificity of the 4CCORD survey for detecting COPD, asthma and other CRDs.

**Table S1:** Table of variables included in Lasso regression models

| Q# | Question/Variable | Options | Asthma | COPD |
|---|---|---|---|---|
| | Age | | √ | √ |
| | Country | | √ | √ |
| | BMI | | √ | √ |
| Q1001 | What is the participant's sex? | ❑ Male 01<br>❑ Female 02 | √ | √ |
| Q1007 | Do you usually cough when you don't have a cold?<br>*[If **yes**, continue with Q7A; If **no**, skip to Q1008]* | ❑ Yes 01<br>❑ No 02 | √ | √ |
| Q1007<br>A | Are there months in which you cough on most days?<br>*[If **yes**, ask both Q1007B & Q1007C; If **no**, skip to Q1008]* | ❑ Yes 01<br>❑ No 02 | √ | √ |
| Q1007B<br>Q1007C | Do you cough on <u>most days</u> for as much as <u>three months each year</u>? | ❑ Yes 01<br>❑ No 02 | √ | √ |
| | For how <u>many years</u> have you had this cough? | ❑ Less than 2 years 01<br>❑ 2-5 years 02<br>❑ More than 5 years<br>03<br><br>Less than 2<br>years<br><br>Less than 2 years | √ | √ |

| | | | | |
|---|---|---|---|---|
| Q1008 | Do you usually bring up phlegm from your chest, or do you usually have phlegm in your chest that is difficult to bring up when you don't have a cold?<br>[If **yes**, continue with Q1008A; If **no**, skip to Q1009] | ❑ Yes 01<br>❑ No 02 | √ | √ |
| Q1008 A | Are there months in which you have this phlegm on most days?<br>[If **yes,** ask both Q1008B & 8C; If **no**, skip to Q1009] | ❑ Yes 01<br>❑ No 02 | √ | √ |
| Q1008B | Are there months in which you have this phlegm on most | | | |
| Q1008C | Do you bring up this phlegm on most days for as much as three months each year? | ❑ Yes 01<br>❑ No 02 | √ | √ |
| | For how many years have you had this phlegm? | ❑ Less than 2 years 01<br>❑ 2-5 years 02<br>❑ More than 5 years 03 | √ | √ |
| Q1009 | Have you had wheezing or whistling in your chest at any time in the last 12 months?<br>*[If **yes**, ask both Q1009A & Q1009B; If **no**, skip to Q1010]* | ❑ Yes 01<br>❑ No 02 | √ | √ |
| Q1009 A | In the last 12 months, have you had this wheezing or whistling only when you have a cold? | ❑ Yes 01<br>❑ No 02 | √ | √ |
| Q1009B | In the last 12 months, have you ever had an attack of wheezing or whistling that has made you feel short of breath? | ❑ Yes 01<br>❑ No 02 | √ | √ |
| Q1010 | Do you ever have trouble with your breathing?<br>*[If **yes**, go to Q1010A or else go to Q1011]* | ❑ Yes 01<br>❑ No 02 | √ | √ |

| | | | | |
|---|---|---|---|---|
| Q1010A | Do you have this trouble? | ❑ Continuously so that your breathing is never, quite right? 01<br>❑ Repeatedly, but it always gets completely better? 02<br>❑ Only rarely? 03 | √ | √ |
| Q1011 | Are you unable to walk due to a condition other than shortness of breath?<br>[*If **yes** to Q1011, please describe this condition on the line below and then skip to Q1013. If **no*** or unsure*, go directly to Q1012* | ❑ Yes 01<br>❑ No 02 | √ | √ |
| Nature of condition(s): --------------------------------------------------------------- | | | | |
| Q1013 | Have you woken up with a feeling of tightness in your chest at any time in the last **12 months**? | ❑ Yes 01<br>❑ No 02 | √ | √ |
| Q1014 | Have you had an attack of shortness of breath that came on during the day when you were at rest at any time in the last ***12 months***? | ❑ Yes 01<br>❑ No 02 | √ | √ |
| Q1015 | Have you had an attack of shortness of breath that came on ***following*** strenuous activity at any time in the last ***12 months***? | ❑ Yes 01<br>❑ No 02 | √ | √ |
| Q1016 | Have you been woken by an attack of shortness of breath at any time in the last ***12 months?***<br>*[If **yes**, go to Q 1016A, else go to Q1017]* | ❑ Yes 01<br>❑ No 02 | √ | √ |
| Q1016A | Have you been woken by an attack of shortness of breath in the last ***3 months***?<br>*[If **yes**, go to Q1016B, else go to Q1017]* | ❑ Yes 01<br>❑ No 02 | √ | √ |

| | | | | |
|---|---|---|---|---|
| Q1016B | ***On average*** have you been woken by an attack of shortness of breath ***at least once a week*** *in the* ***last 3 months?***<br>*[If* ***yes****, go to Q1016C, else go to Q1017]* | ❑ Yes 01<br>❑ No 02 | √ | √ |
| Q1016C | How many times a week **on average** have you been woken by shortness of breath in the **last 3 months**? | ____ ____ ____ times | √ | √ |
| Q1017 | Have you been woken by an attack of coughing at any time *in the last* ***12 months***? | ❑ Yes 01<br>❑ No 02 | √ | √ |
| Q1018 | Has a doctor or other health care provider ever told you that you have emphysema? | ❑ Yes 01<br>❑ No 02 | √ | √ |
| Q1019 | Has a doctor or other health care provider ever told you that you have asthma, asthmatic bronchitis or allergic bronchitis?<br>*[If* ***yes****, ask Q1019A. If* ***no****, skip to Q1020]* | ❑ Yes 01<br>❑ No 02 | √ | √ |
| Q1019A | Do you still have asthma, asthmatic bronchitis or allergic bronchitis? | ❑ Yes 01<br>❑ No 02 | √ | √ |
| Q1020 | Has a doctor or other health care provider ever told you that you have chronic bronchitis?<br>*[If* ***yes****, ask Q1020A. If* ***no****, skip to Q1021]* | ❑ Yes 01<br>❑ No 02 | √ | √ |
| Q1020A | Do you still have chronic bronchitis? | ❑ Yes 01<br>❑ No 02 | √ | √ |
| Q1021 | Has a doctor or other health care provider ever told you that you have chronic obstructive pulmonary disease (COPD)?<br>*[If* ***yes****, ask Q1021A. If* ***no****, skip to Q1022]* | ❑ Yes 01<br>❑ No 02 | √ | √ |
| Q1021A | *Do you still have COPD?* | ❑ Yes 01<br>❑ No 02 | √ | √ |

| | | | | |
|---|---|---|---|---|
| Q1023 | Have you ever had a period when you had breathing problems that got so bad that they interfered with your usual daily activities or caused you to miss work?<br>*[If **yes**, ask Q1023A. If **no**, skip to Q1024]* | ❑ Yes 01<br>❑ No 02 | √ | √ |
| Q1023A | How many such episodes have you had in the past 12 months?<br>*[If Q1023A >0, ask Q1023B and Q1023C, else skip to Q1024]* | ____ ____ ____ episodes | √ | √ |
| Q1023B | For how many of these episodes did you need to see a doctor or other health care provider in the past 12 months? | ___ ____ ____ episodes | √ | √ |
| Q1023C | For how many of these episodes were you hospitalized overnight in the past 12 months?<br>*[If Q1023C >0, ask Q123D, else skip to Q1024]* | ____ ____ ____ episodes | √ | √ |
| Q1023D | Altogether, for how many total days were you hospitalized overnight for breathing problems in the past 12 months? | ____ ____ ____ days | √ | √ |
| Q1028 | Have you <u>ever</u> smoked cigarettes? | ❑ Yes 01<br>❑ No 02 | √ | √ |
| | *("Yes," means more than 20 packs of cigarettes in a lifetime or more than 1 cigarette each day for a year) [if yes, ask Q1028A through Q1028D; otherwise, skip to Q1030]* | | | √ |
| Q1028A | How old were you when you first started regular cigarette smoking? | _____ _____ years old | √ | √ |
| Q1028B | <u>If you have stopped smoking</u>, how old were you when you last stopped? (If the participant has not stopped smoking, record as code '99'.) | _____ _____ years old | √ | √ |
| Q1028C | On average over the entire time that you smoke(d), about how many cigarettes per day do (did) you smoke? | ____ ____ ____ cigarettes/day | √ | √ |

| | | | | |
|---|---|---|---|---|
| Q1028D | On average over the entire time that you smoke(d), do (did) you primarily smoke manufactured or hand-rolled cigarettes? | ❑ Manufactured 01<br>❑ Hand-rolled 02 | √ | √ |
| Q1030 | Have you <u>ever</u> smoked a pipe or cigar?<br>*[If **yes**, ask Q1030A. If **no**, proceed to Q1031]* | ❑ Yes 01<br>❑ No 02 | √ | √ |
| Q1030A | Do you now smoke a pipe or cigar? | ❑ Yes 01<br>❑ No 02 | √ | √ |
| Q1033 | Have you been **regularly** exposed to tobacco smoke in the last ***12 months***?<br>*['Regularly' means on most days or nights]?*<br>*[If **no**, go to Q1034, If **yes** Q1033A]* | ❑ Yes 01<br>❑ No 02 | √ | √ |
| Q1033A | Not counting yourself, how many people in your household smoke regularly? | ____ ____ ____ number | √ | √ |
| Q1033B | Do people smoke regularly in the room where you work? | ❑ Yes 01<br>❑ No 02 | √ | √ |
| Q1033C | How many hours per day are you exposed to ***other people's*** tobacco smoke? | ____ ____ hours | √ | √ |
| Q1033D | How many hours per day, are you exposed to other people's tobacco smoke in the following locations?<br>a) at home,<br>b) workplace,<br>c) bars, restaurants, cinemas or similar social settings<br>d) elsewhere | <br><br><br>____ ____ hours<br>____ ____ hours<br>____ ____ hours<br>____ ____ hours | √ | √ |
| Q1034 | Have you ever worked for a year or more in a dusty job?<br>*[If **yes**, ask Q1034A, otherwise skip to Q1035]* | ❑ Yes 01<br>❑ No 02 | √ | √ |
| Q1034A | For how many years have you worked in dusty jobs? | _____ _____ years | √ | √ |

| | | | | |
|---|---|---|---|---|
| Q1036 | Has a doctor or other health care provider ever told you that you had the following? | | | √ |
| Q1036A | Heart disease | ❑ Yes 01<br>❑ No 02 | √ | √ |
| Q1036B | Hypertension | ❑ Yes 01<br>❑ No 02 | √ | √ |
| Q1036C | Diabetes | ❑ Yes 01<br>❑ No 02 | √ | √ |
| Q1036D | Lung cancer | ❑ Yes 01<br>❑ No 02 | √ | √ |
| Q1036E | Stroke | ❑ Yes 01<br>❑ No 02 | √ | √ |
| Q1036F | Tuberculosis<br>*[If **yes** to Q1036F, then ask Q1036F1; otherwise, skip to Q1036G]* | ❑ Yes 01<br>❑ No 02 | √ | √ |
| Q1036F1 | Are you currently taking medicine for tuberculosis?<br>*[If **no** to Q1036F1, then ask Q1036F2; otherwise, skip to Q1036G]* | ❑ Yes 01<br>❑ No 02 | √ | √ |
| Q1036F2 | Have you ever taken medicine for tuberculosis? | ❑ Yes 01<br>❑ No 02 | √ | √ |
| Q1036G | Nasal allergies, including hay fever? | ❑ Yes 01<br>❑ No 02 | √ | √ |
| Q1036H | Eczema? | ❑ Yes 01<br>❑ No 02 | √ | √ |
| Q1036I | Gastro-oesophageal reflux? | ❑ Yes 01<br>❑ No 02 | √ | √ |
| Q1037 | Have you ever had an operation on your chest in which a part of your lung was removed? | ❑ Yes 01<br>❑ No 02 | √ | √ |

| | | | | |
|---|---|---|---|---|
| Q1038 | Were you hospitalized as a child for breathing problems <u>prior to</u> the age of 10? | ❑ Yes 01<br>❑ No 02<br>❑ Don't Know 03 | √ | √ |
| Q1040 | Has a doctor or other health care professional told your father, mother, sister or brother that they had a diagnosis of emphysema, chronic bronchitis or COPD? | ❑ Yes 01<br>❑ No 02 | √ | √ |
| Q1041 | Has a doctor or other health care professional told your father, mother, sister or brother that they had a diagnosis of eczema, asthma or hay fever? | ❑ Yes 01<br>❑ No 02 | √ | √ |
| Q1042 | Has anyone living in your home (besides yourself) smoked a cigarette, pipe or cigar in your home during the past two weeks? | ❑ Yes 01<br>❑ No 02 | √ | √ |
| Q1043 | In general, would you say your health is: *[Check one.]* | ❑ Excellent 01<br>❑ Very good 02<br>❑ Good 03<br>❑ Fair 04<br>❑ Poor 05 | √ | √ |
| Q1057 | Has an indoor open fire with wood, crop residues or dung been used as a primary means of cooking in your home for more than 6 months in your life?<br>*[If **yes**, ask Q1057A to Q1057D, else skip to Q1058]* | ❑ Yes 01<br>❑ No 02 | √ | √ |
| Q1057A | For how many years have wood, crop residues or dung been used for cooking in your home? | ______ ______ years | √ | √ |
| Q1057B | On average, for how many hours a day have you personally spent cooking using wood, crop residues or dung? | ______ ______ hours | √ | √ |

| | | | | |
|---|---|---|---|---|
| Q1057C | Are wood, crop residues or dung still used for cooking in your home? | ❑ Yes 01<br>❑ No 02 | √ | √ |
| Q1057D | Was (Is) your stove or fire vented to the outside (e.g., through a chimney or window)? | ❑ Yes 01<br>❑ No 02 | √ | √ |
| Q1058 | Have you used an open fire with coal or coke as a primary means of heating your home for more than 6 months in your life?<br>*[If **yes**, ask Q1058A and Q1058B, else skip to Q1059]* | ❑ Yes 01<br>❑ No 02 | √ | √ |
| Q1058A | For how many years have you used an open fire with coal or coke as a primary means of heating your home? | ______ ______ years | √ | √ |
| Q1058B | Do you still use an open fire with coal or coke as a primary means of heating your home? | ❑ Yes 01<br>❑ No 02 | √ | √ |
| Q1059 | Have you used an open fire with wood, crop residues or dung as a primary means of heating your home for more than 6 months in your life?<br>*[If **yes**, ask Q1059A and Q1059B, else skip to end of questionnaire]* | ❑ Yes 01<br>❑ No 02 | √ | √ |
| Q1059A | For how many years have you used an open fire with wood, crop residues or dung as a primary means of heating your home? | ______ ______ years | √ | √ |
| Q1059B | Do you still use an open fire with wood, crop residues or dung as a primary means of heating your home? | ❑ Yes 01<br>❑ No 02 | √ | √ |
| | pre_FEV1 | | √ | √ |
| | pre_FEV1_pred | | √ | √ |
| | pre_FEV1/FVC | | √ | √ |
| | pre_FVC | | √ | √ |
| | pre_FVC_pred | | √ | √ |

| | | | | |
|---|---|---|---|---|
| | post_FEV1 | | √ | √ |
| | post_FEV1_pred | | √ | √ |
| | post_FEV1/FVC | | √ | √ |
| | post_FVC | | √ | √ |
| | post_FVC_pred | | √ | √ |
| | Reversibility | | √ | |

**Table S2.** Table of the prevalence of asthma, COPD, restrictive spirometry (based on GLI predictive values) and 'other CRD' at the five sites based on consensus categorization.

| Gold Standard Diagnosis | Bangladesh | CMC | KEMHRC | Malaysia | Pakistan | Total |
|---|---|---|---|---|---|---|
| COPD | 7 | 3 | 2 | 3 | 8 | 23 |
| Asthma (spirometry) | 1 | 0 | 1 | 2 | 4 | 8 |
| Asthma (symptoms) | 13 | 9 | 6 | 19 | 28 | 75 |
| Other CRD | 1 | 0 | 0 | 9 | 5 | 15 |
| RLD | 14 | 13 | 16 | 13 | 9 | 65 |
| No CRD | 47 | 44 | 50 | 32 | 22 | 195 |
| Isolated symptom (CRD unlikely) | 13 | 7 | 11 | 2 | 9 | 42 |
| Restrictive (asymptomatic) | 3 | 21 | 11 | 8 | 2 | 45 |
| Non-respiratory | 2 | 1 | 7 | 6 | 12 | 28 |
| Unclear | 0 | 2 | 2 | 7 | 1 | 12 |
| **Total** | **101** | **100** | **106** | **101** | **100** | **508** |

RLD: Restrictive Lung Disease
No CRD: No Chronic Respiratory Disease
Non-respiratory: Non-respiratory cause for symptoms
Note: The differences in prevalences between the sites are likely to be due the small sample sizes in this feasibility study rather than real differences in populations that would have been captured in a fully powered survey which would have required approximately 1,000 patients/site.

**Table S3:** Table of parameter estimates for a predictive model for COPD for participants with asthma or COPD

| Parameter | Lasso predictive model estimate |
|---|---|
| *Intercept* | 1.877 |
| Age | 0.040 |
| Pre-FVC%pred | 0.074 |
| Post-FEV1%pred | -0.179 |
| Post-FVC%pred | 0.023 |
